# Anterior Open Bite Treated With Palatal Crib and Myofunctional Therapy: A Case Report

**DOI:** 10.7759/cureus.63549

**Published:** 2024-06-30

**Authors:** Rohini Chandel, Meenal S Pande, Ramakrishna Yeluri, Neha Pankey, Monika Khubchandani

**Affiliations:** 1 Pediatric Dentistry, Sharad Pawar Dental College and Hospital, Datta Meghe Institute of Higher Education and Research (Deemed to be University), Wardha, IND

**Keywords:** overbite, tongue thrusting habit, thumb sucking habit, anterior open bite, myofunctional treatment, palatal crib

## Abstract

The most prevalent oral habit and one of the most often habitual behavioral patterns in preschool-aged children is thumb-sucking. This behavior is crucial to the development of malocclusion and must be addressed carefully since it may cause a secondary tongue thrust that worsens the issue. Developing an effective treatment plan requires determining the underlying cause, which may include psychological, physiological, and or anatomical factors. Overall prevention of behavior needs to be planned for successful outcomes. One such device for treating tongue-thrusting and thumb-sucking habits is the palatal crib. The present case shows the possible effectiveness of palatal crib use in conjunction with myofunctional therapy for a child whose diagnosis involves habitually holding the tongue low and sucking the thumb that causes an anterior open bite (AOB). An 11-year-old boy with flared and spaced upper and lower incisors also had an AOB. Myofunctional therapy was combined with palatal cribs to help the tongue reposition itself and discourage the habit of sucking. The AOB was successfully corrected with an appropriate overjet and overbite after a total of three months of treatment.

## Introduction

Habits are defined as repetitive and automatic behaviors that are performed regularly [1]. The main way we communicate our emotions is through our mouths. It also decreases desire and anxiety in both adults and children. When the area is stimulated with the tongue, finger, or sometimes a nail, it produces a relaxing sensation [2]. From the time they initially develop at 29 weeks of age, these behaviors continue until adulthood in the permanent, mixed, and deciduous dentition. Throughout the mixed dentition, if the tendency continues, malocclusion may develop [3].

Numerous circumstances such as an unfavorable growth pattern, finger, and pacifier-sucking behaviors, retained swallowing patterns, swollen lymph nodes, tongue position, and function from infancy might result in an anterior open bite (AOB). Because of this, it is difficult to treat such complex malocclusions. A pediatric dentist will typically need to combine dentofacial orthopedic treatment, orthodontics, and behavior modification [4].

A persistent habit of sucking one's thumb causes the lips to become incompetent, the maxillary incisors to protrude, the lip seal necessary for swallowing to be broken, and ultimately an AOB [5]. The tongue may, however, be positioned abnormally when swallowing or at rest in conjunction with the thumb-sucking behavior, which could exacerbate an AOB. The main cause of the AOB relapse may be the inability to adjust tongue posture [6].

In addition to helping with AOB correction, the palatal crib keeps the tongue from resting on the upper teeth. The palatal crib, on the other hand, might enable the tongue to reposition itself low preventing the tongue from being functionally re-educated and ultimately leading to an AOB recurrence [7]. In this instance, realigning and retraining the tongue to its natural position will require myofunctional therapy. Myofunctional therapy is a combination of physical therapy. Considering it as a physical therapy, designed specifically for the face, tongue, and muscles of the mouth, the exercises are made expressly for improving speaking, breathing, chewing, and swallowing correctly.

The following case report will show how palatal crib therapy, in conjunction with myofunctional therapy, helped a child who exhibited habitual anterior and low tongue postures as well as thumb-sucking.

## Case presentation

**Figure 1:**
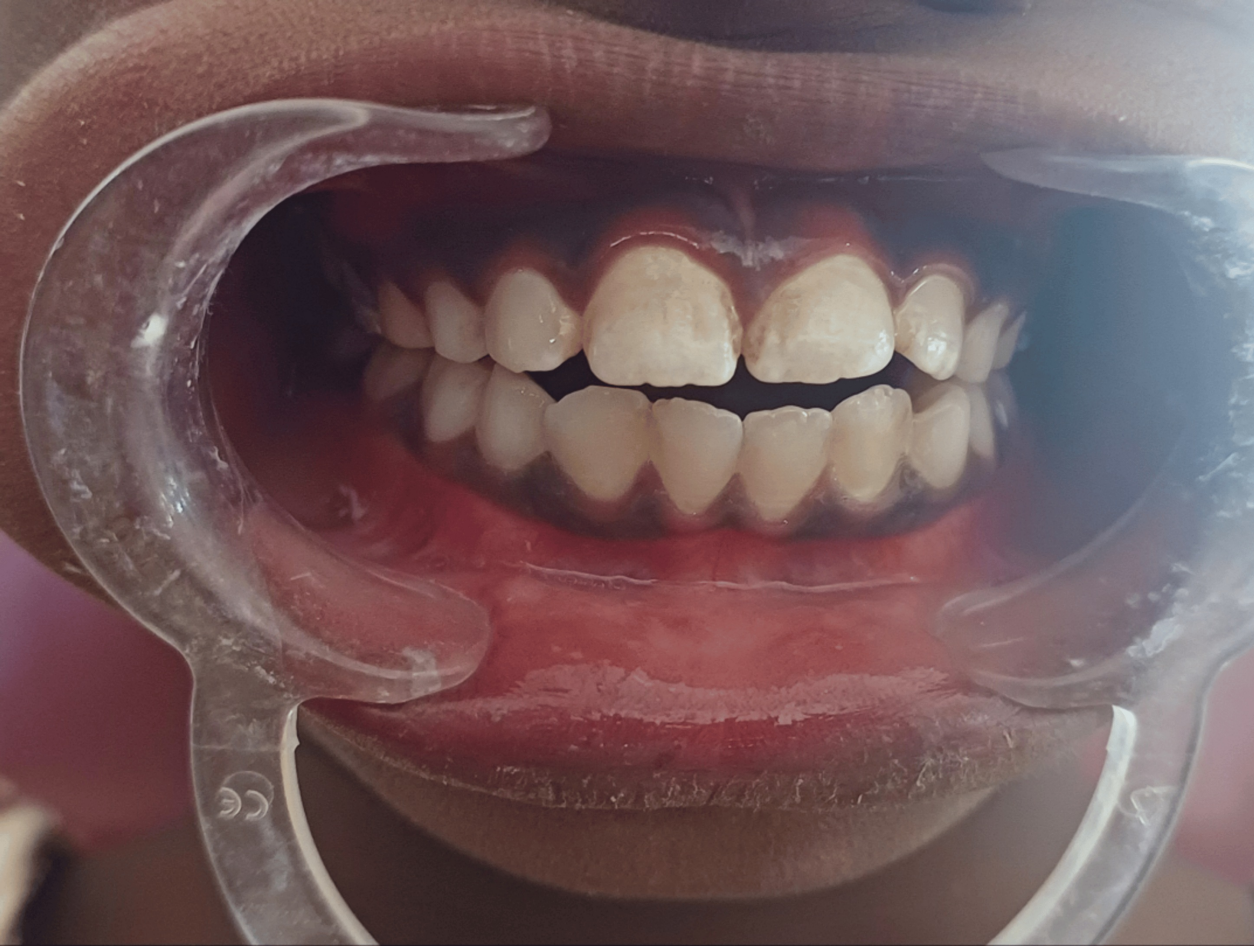
Preoperative photograph showing an anterior open bite This figure shows that the child has an anterior open bite which denotes a lack of contact between the upper and lower incisors while the posterior teeth are in class I molar relationship bilaterally. Here, the spacing between the upper and lower central incisors is 4 mm and continues laterally to the canine on both sides. Due to prolonged thumb-sucking, the child developed an anterior open bite.

In the Department of Pedodontics and Preventive Dentistry, an 11-year-old child and his father were reported with a main complaint of thumb-sucking habit since he was two years old. After a thorough medical history was taken, the father disclosed that the child routinely spends 7-8 hours a day during waking hours sucking his thumb. The results of the intraoral examination show a mixed dentition stage with a right and left class I molar relationship and an open bite measuring 4 mm at the central incisors and continuing laterally to the canines on both sides. Due to the prolonged and severe thumb-sucking, the child exhibited an AOB along with simple tongue pushing (Figure 1).

Two simultaneous strategies were used in the plan: A fixed device for breaking habits, such as a palatal crib, which served to guide the tongue into a natural resting position and inhibit the practice of sucking. The second strategy involved performing daily myofunctional exercises under parental supervision at home to adjust the tongue to its usual resting position. One of the exercises involved inserting the tip of the tongue behind the cribs in the front region of the palate and then pushing down firmly to create a popping sound. Similar to the previous exercise, the second involved pressing the tongue tip upward rather than placing it in the front region of the palate. Each exercise was to be performed by the patient at least 10 times.

**Figure 2:**
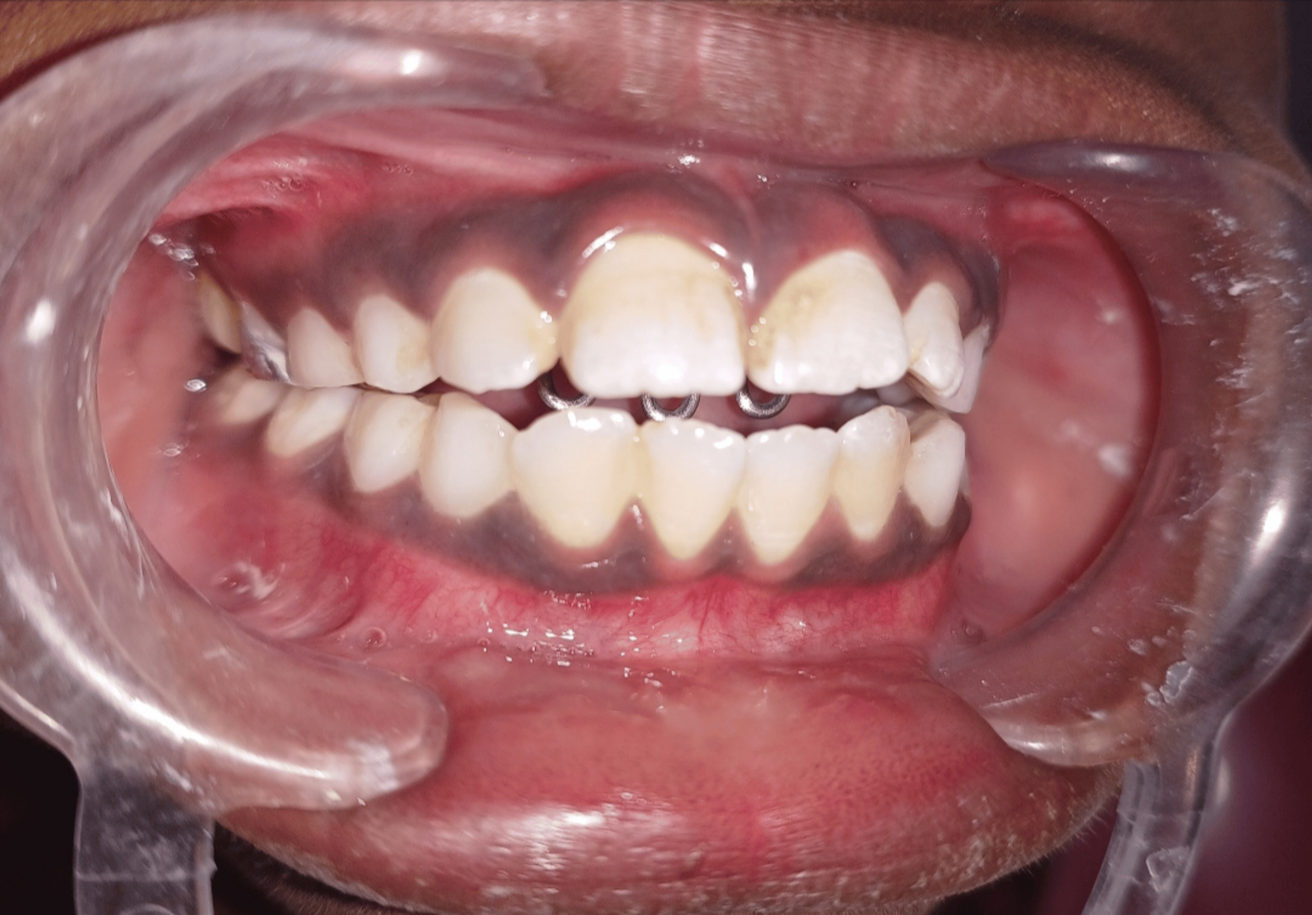
Intraoral photograph showing palatal crib insertion This figure shows that the palatal crib was inserted in the oral cavity of the child on the subsequent visit. The restricted movements of the tongue were seen behind the palatal crib.

After banding the primary molars, an alginate impression was created. Using 0.8 mm stainless steel wire, the crib was constructed on the mold. The crib's joints were soldered, and tiny round beads were set on top of the crib using silver solder. Glass ionomer cement (GIC) type I was used to perform the appliance cementation at the following visit (Figure 2).

**Figure 3:**
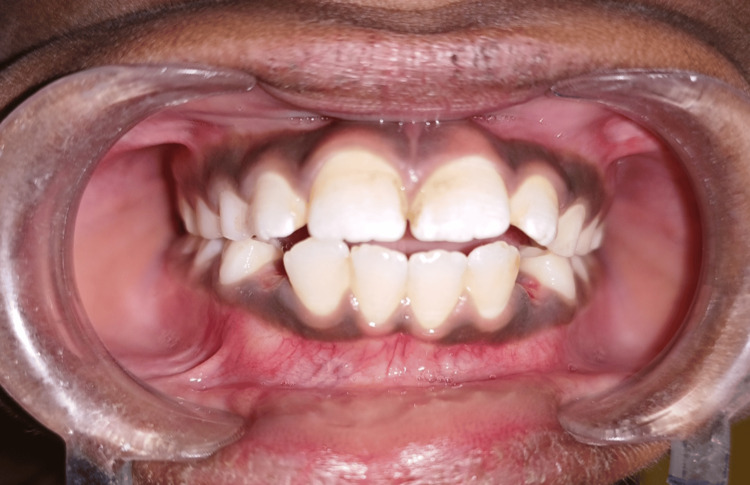
Photograph showing one-month follow-up after crib insertion An anterior open bite was decreased after one month following the insertion of the palatal crib. Deciduous canine is seen as an exfoliation bilaterally.

**Figure 4:**
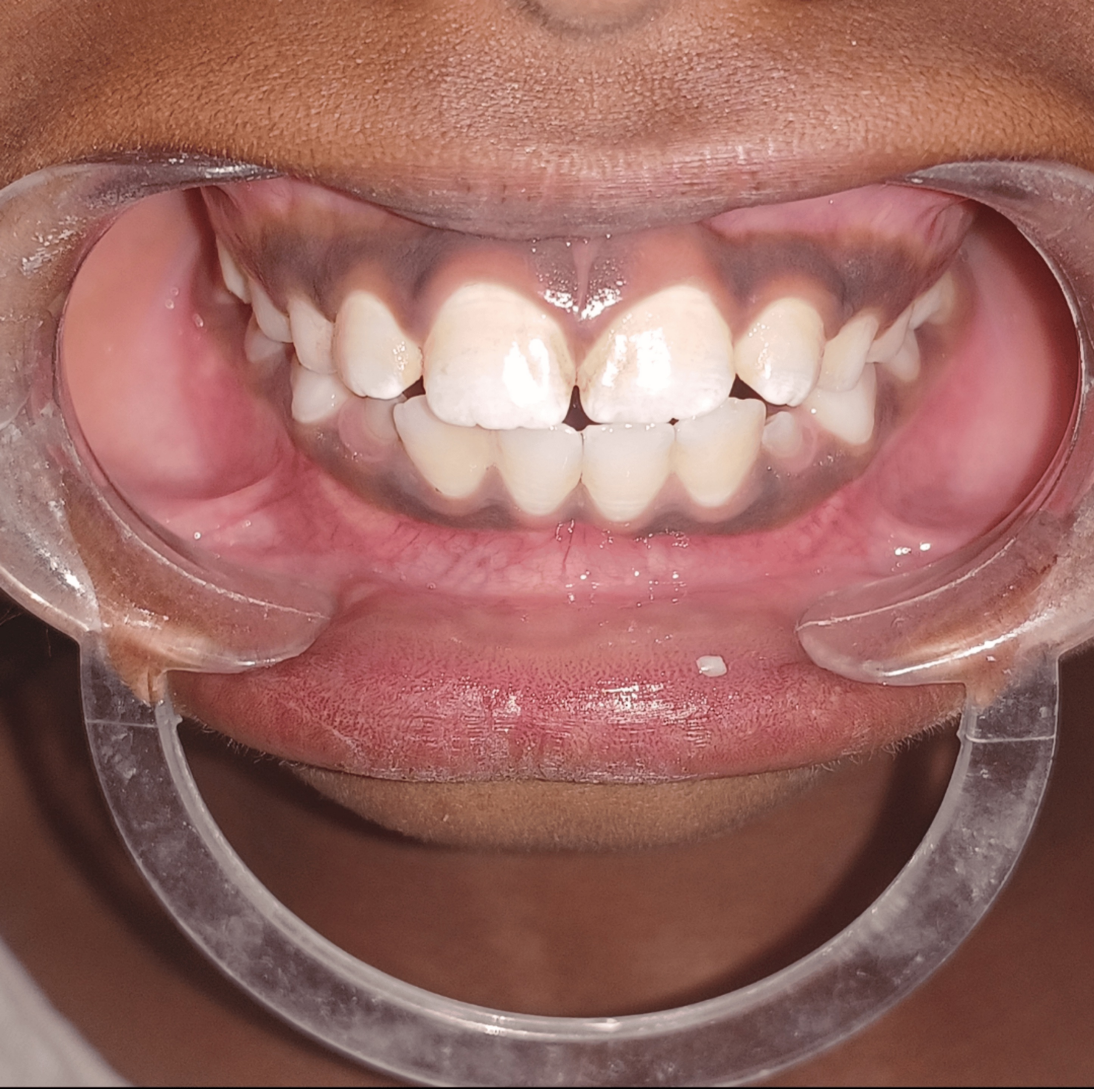
Photograph showing the three-month follow-up after crib insertion This figure shows that the anterior open bite has adequately decreased in three months after the palatal crib was inserted. The closure of the anterior open bite was achieved and the canine erupted in normal occlusion bilaterally

The patient was recalled after two weeks. The positive feedback was noted as told by his father. The habit had significantly decreased at the three-month follow-up examination (Figures 3-4).

The patient was instructed to wear an appliance following habit reversal for at least three months, but their posttreatment follow-up showed no signs of relapse. The patient was advised to continue the myofunctional activities for a minimum of five months after removing the device in order to maintain the stability of the therapeutic effects required.

## Discussion

The patient completed palatal crib therapy in just three months, and then he continued his myofunctional exercises for five more months. This case study demonstrates that the palatal crib can effectively prevent thumb-sucking behavior. The AOB was significantly corrected after three months, and myofunctional treatment was beneficial in helping the patient adopt a normal resting tongue position and preserve the stability of the AOB correction.

It is believed that the behavior of sucking one's finger (or thumb) is carried out for psychological comfort and oral enjoyment. Severe digit sucking can result in mandibular incisor retroclination and maxillary anterior proclination, constriction of the maxilla, increased overjet, and AOB [8].

AOB brought on by thumb-sucking typically results in a secondary tongue push, which exacerbates the issue. Positive reinforcement, encouraging the patient to break the habit, equipment that functions as a mechanical barrier, and tactile reminders are all part of the treatment plan for extended digit sucking. Crib appliances in the anterior region are proven to be quite useful both as physical restraints and as reminders [8].

In this instance, the patient also had a tongue-pushing tendency in addition to thumb-sucking. It's critical to comprehend the etiology, which encompasses psychological, physiological, and anatomical factors, to establish an effective course of treatment. Eradication of behavior is planned for the desired results.

The palatal crib corrects AOB by preventing the tongue from resting on the maxillary incisors and discouraging thumb-sucking. However, in certain instances, a palatal crib may not be sufficient to control and adapt tongue position; hence, it is recommended to combine it with myofunctional therapy to aid the tongue in returning to its normal position. The control of the main causes of AOB is the foundation for the stability of the AOB correction [9]. Furthermore, Dias hypothesized that the inability to modify tongue posture may be the main cause of AOB relapse [6]. In addition, Smithpeter and Covell examined the effectiveness of myofunctional therapy in maintaining the closure of open bites in conjunction with orthodontic treatment and discovered that the patients with forward tongue posture and tongue thrust, myofunctional therapy increased the stability of AOB correction [10]. According to Heo, in cases with AOB, the 3D position of maxillary incisors should be taken into consideration while determining the best tooth movement pattern to accomplish facial aesthetics [11]. In addition, Zawawi said that it is crucial to maintain a balance between the functional and aesthetic requirements while developing and implementing the selected approach of correction. It was necessary to enhance the incisal display while preventing additional alveolar bone resorption. A grin that is both beautiful and appealing has a consonant smile arc and 2 mm of gingival display [12].

## Conclusions

When an AOB is present, abnormal tongue position and habits must be properly understood and corrected. Before utilizing tools that disrupt habits, it is advised to use the least intrusive techniques, such as counseling. Appliances designed to break habits are recommended for children who require extra help to break them. Appliances that break bad habits can be mended or taken apart. Breaking harmful oral habits decreases the likelihood of problems with the development of the facial bones and the positioning of the teeth. An appliance that is used permanently to help break the habit is the palatal crib. Myofunctional therapy combined with a palatal crib is successful in treating AOB caused by low and anterior tongue position and thumb-sucking habits. Furthermore, the myofunction therapy improved the stability of the open bite correction and offered improved control over tongue position.
